# The crucial importance of preventive and cardiac rehabilitation programmes in patients with atrial fibrillation: AF-CARE units

**DOI:** 10.1093/europace/euaf016

**Published:** 2025-01-17

**Authors:** Ercan Akşit, Uğur Küçük, Gökay Taylan

**Affiliations:** Department of Cardiology, Canakkale Onsekiz Mart University Faculty of Medicine, Canakkale, Turkey; Department of Cardiology, Canakkale Onsekiz Mart University Faculty of Medicine, Canakkale, Turkey; Department of Cardiology, Trakya University Faculty of Medicine, Edirne, Turkey

We read with great interest the article by Cheng *et al*.^1^ in which they stated that the prevalence of atrial fibrillation (AF) increased by 1.37 times and its incidence by 1.24 times in 31 years. It is very important to establish specific units that will provide both preventive health services and follow up patients for AF, which is increasingly becoming a major public health problem. They emphasized that because AF is preventable and treatable, cost-effective techniques targeting modifiable risk factors should be urgently adopted in regions with increasing prevalence rates.^1^ Walli-Attaei *et al*.^2^ highlighted the significant burden, which AF-related symptoms and cardiovascular events (CVEs) impose on both patients and healthcare systems, particularly healthcare costs, in Europe and Central Asia. The latest published consensus article stated that patients with AF and low arrhythmia burden have a lower risk of stroke and other CVEs than those with high arrhythmia burden, and compiled what needs to be done.^3^ Salmela *et al*.^4^ emphasized that women use antiarrhythmic drugs more than men, but they undergo ablation and cardioversion procedures less than those younger than 65 years of age. Zörner *et al*.^5^ reported in their study that older patients, women, patients with lower levels of education were less likely to receive AF ablation. Okoye *et al*.^6^ noted that older adults with AF exhibited a more rapid annual decline in walking speed compared with those without AF in their 15-year follow-up study. While high body mass index (BMI) is a known risk factor for the development of AF, Vermeer *et al*.^7^ emphasized that obesity is an independent risk factor for repeat ablation. The latest guideline has provided us with the most systematic approach to AF patients to date.^8^ The application of AF-*CARE* (*C*omorbidity and risk factor management, *A*void stroke and thromboembolism, *R*educe symptoms by rate and rhythm control, *E*valuation and dynamic reassessment) principles to all patients is emphasized.^8^ Additionally, new opportunities in technology (especially innovations in artificial intelligence and genetics) may provide cardiologists with new guidance to predict AF, improve AF screening, and personalize patient management.^9^ Finally, colchicine has been shown by meta-analysis to prevent the development of AF in postoperative patients.^10^ An AF-CARE unit can be established in all cardiology clinics, starting with preventive cardiology services for each individual (e.g. maintaining a BMI of 20–25 kg/m^2^, 150–300 min of moderate-intensity aerobic physical exercise per week, population-based screening using a prolonged non-invasive ECG-based approach to enable earlier detection of AF^8^ in individuals aged ≥75 years or ≥65 years with additional risk factors), and continuing with cardiac rehabilitation programmes for all AF patients, whether or not they have had ablation.

In conclusion, AF-CARE (including both prevention and rehabilitation programmes) units may contribute to reducing mortality and morbidity in AF patients by using current guidelines and new information (e.g. 3–10) in primary and secondary prevention of the disease.
